# A Novel Adaptive Independent Component Analysis Method for Multi-Channel Optically Pumped Magnetometers’ Magnetocardiography Signals

**DOI:** 10.3390/bios15040243

**Published:** 2025-04-11

**Authors:** Shuang Liang, Jiahe Qi, Junhuai He, Yikang Jia, Aimin Wang, Ting Zhao, Chaoliang Wei, Hongchen Jiao, Lishuang Feng, Heping Cheng

**Affiliations:** 1 School of Instrumentation Science and Optoelectronics Engineering, Beihang University, Beijing 100191, China; sliang0607@gmail.com (S.L.); qi1183663165@163.com (J.Q.); hjh2220000@gmail.com (J.H.); jyk8161082022@163.com (Y.J.); 2 School of Electronics, Peking University, Beijing 100871, China; wangaimin@pku.edu.cn; 3 PKU-Nanjing Institute of Translational Medicine, Nanjing Raygen Health, Nanjing 210031, China; zhaoting@raygenitm.com (T.Z.); chaoliangwei@126.com (C.W.); 4 Beijing Laboratory of Biomedical Imaging, Beijing Municipal Education Commission, Beijing 100085, China; 5 National Biomedical Imaging Center, State Key Laboratory of Membrane Biology, Peking-Tsinghua Center for Life Sciences, College of Future Technology, Peking University, Beijing 100871, China

**Keywords:** magnetocardiography, optically pumped magnetometers, biosensors, bio-signal process, independent component analysis

## Abstract

With the gradual maturation of optically pumped magnetometer (OPM) technology, the use of OPMs to acquire weak magnetocardiography (MCG) signals has started to gain widespread application. Due to the complexity of magnetic environments, MCG signals are often subject to interference from various unknown sources. Independent component analysis (ICA) is one of the most widely used methods for blind source separation. However, in practical applications, the numbers of retained components and filtering components are often selected manually, relying on subjective experience. This study proposes an adaptive ICA method that estimates the signal-to-noise ratio (SNR) before processing to determine the number of components and selects heartbeat-related components based on their characteristic indicators. The method was validated using phantom experiments and MCG data in a 128-channel OPM-MCG system. In the human subject experiment, the array output SNR reached 31.8 dB, and the processing time was significantly reduced to 1/38 of the original. The proposed method outperformed traditional techniques in terms of its ability to identify artifacts and efficiency in this regard, providing strong support for the broader clinical application of OPM-MCG.

## 1. Introduction

Magnetocardiography (MCG) is a new medical imaging technique that measures the extremely weak magnetic fields generated by the activation currents along myocardial fibers during heart activity [1,2]. The sensitivity of MCG in detecting ischemic myocardium in both its resting and activated states is superior to that of electrocardiography (ECG) [3,4,5], and as a complementary diagnostic tool to ECG, it has gradually found use in clinical trials.

Some researchers have studied MCG using superconducting quantum interference devices due to the advantages of MCG [6,7]. However, with the advent of the spin-exchange relaxation-free (SERF) OPM technology, which does not rely on low-temperature cooling, significant potential has been demonstrated [8]. OPM sensors have low operational costs (no liquid helium consumption) and offer flexible layout options, providing higher-quality data [9,10,11]. This breakthrough could drive the development of MCG technology and further expand its application scenarios [12].

The detectable amplitude of magnetic heart signals on the body’s surface is in the range of tens of pT, while the Earth itself, as a giant magnet, has a magnetic field amplitude of tens to hundreds of μT, which is approximately seven orders of magnitude larger than the heart’s magnetic field [13]. In urban or indoor environments, the magnetic field characteristics of the surroundings are more complex. The movement of vehicles, subways, indoor elevators, and the use of alternating current electricity from ferromagnetic objects all contribute to fluctuations in the magnetic field [14]. These forms of magnetic noise and interference present significant challenges to the detection and extraction of magnetic heart signals.

Independent component analysis (ICA) is a statistical method used to separate multivariate signals into statistically independent sub-signals, and it has been widely applied in multi-channel MCG signal processing to separate signals from different sources (e.g., magnetic heart signals and various noise sources) [15,16,17,18]. However, the identification of whether the separated independent components are noise or artifacts often relies on empirical judgment, and manual selection is required to eliminate artifact components [19]. This dependence on experience not only makes the data processing difficult to quantify but also increases the time required for signal processing, making the clinical application of magnetocardiography challenging [20,21].

To overcome this challenge in OPM-MCG signal processing, this study proposes an adaptive ICA signal processing scheme that combines the autocorrelation function interval power ratio (IPR) and cross-correlation function extremum (CFE) to select and filter components. The proposed scheme simplifies the OPM-MCG signal processing workflow and reduces reliance on human experience.

To validate the efficacy of the proposed method, a series of phantom simulation experiments and real signal experiments were conducted to evaluate both the effectiveness and processing efficiency of the method. Initially, simulated heartbeat signals were generated and combined with various noise signals (including band-limited white noise and power line interference). The coefficients of the correlation between the original signal and the denoised signal processed by the proposed method were calculated to assess the ability to recover heartbeat signals. Furthermore, the proposed processing flow was applied to denoise the signals for real-subject tests using the OPM-MCG for heartbeat signal acquisition. The resulting multi-channel single-cycle MCG signal butterfly plots were generated and verified, demonstrating the effectiveness of the algorithm in real-world MCG acquisition and processing applications.

## 2. Materials and Methods

### 2.1. ICA Dimensionality Reduction

For spatially distributed sensors, the process through which source signals propagate to the sensors can be expressed as follows:

(1) X=AS

where X is the measurement data matrix, consisting of a matrix with $N_{c}$ channels and T discrete measurement data points in OPM-MCG measurements. S represents the source signal matrix, which in the case of magnetic heart measurements includes the heart’s electrophysiological sources (the signals to be extracted) and potential noise sources, with dimensions of $N_{s}\times T$, where $N_{s}$ is the number of source signals. A is the mixing matrix, representing the conduction matrix that transfers signals from the $N_{s}$ source signals to the $N_{c}$ measuring sensors. The mixing matrix produces a reduced-rank mixture of the noise and signal sources.

ICA works by decomposing the measurement data matrix X. This process is expressed as follows:

(2) $$C=W\hat{X}$$

where W is the decomposition matrix obtained through principal component analysis (PCA) after whitening and dimensionality reduction. The matrix W is a square matrix whose order depends on the number of rows in the matrix X after PCA whitening. The resulting matrix C represents the statistically independent components. Using PCA to reduce the dimensionality of the data, the number of effective dimensions can be estimated through the cumulative variance contribution rate.

(3) $${\mathrm{Var}}_{\mathrm{contribution}}=\frac{\sum_{i=1}^{k}\sigma_{i}^{2}}{\sigma_{\mathrm{total}}^{2}}$$

The variance of the *i*th principal component is represented as $\sigma_{i}^{2}$, and the $\sigma_{\mathrm{total}}^{2}$ is the sum of the variances of all the components. The cumulative variance contribution rate of the first *k* principal components is the proportion of the total variance.

**Figure 1:**
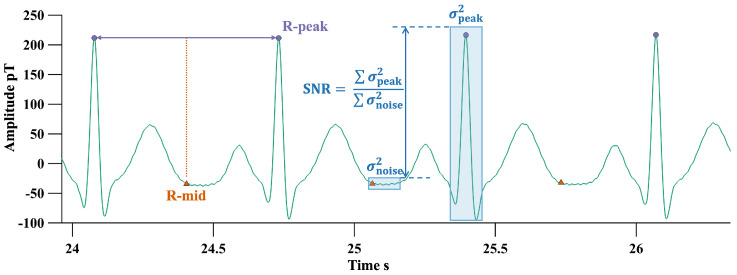
SNR estimation using R-peaks and midpoint between R-peaks.

Due to the uncertainty surrounding the number of measurement equations $N_{c}$ (the number of channels) and the number of sources $N_{s}$, we cannot guarantee that the decomposition process is either underdetermined or overdetermined. As such, not all the components (i.e., each row in C) will have clear physical significance. After dimensionality reduction using PCA, the number of retained components can be estimated based on the cumulative variance contribution rate, but the threshold information still requires manual adjustment based on experience. Therefore, before this process, we perform simple preprocessing on the original signal, segmenting the heartbeats based on the R-wave positions for all channels. We then calculate the power near the R-wave and the midpoint between R-waves for each channel as estimates of the signal and noise power, as shown in Figure 1. The estimated SNR can be calculated as the ratio of the sum of the powers for all *i* channels over *j* heartbeats, denoted as follows:

(4) $$\mathrm{SNR}=\frac{\sum_{i,j}\sigma_{{\mathrm{peak}}_{i,j}}^{2}}{\sum_{i,j}\sigma_{{\mathrm{Noise}}_{i,j}}^{2}}$$

where $\sigma_{{\mathrm{peak}}_{i,j}}^{2}$ represents the power for channel *i* and heartbeat *j* near the R-wave, and $\sigma_{{\mathrm{Noise}}_{i,j}}^{2}$ represents the power near the midpoint between R-waves. Based on this SNR, we estimate the number of principal components to retain.

### 2.2. Autocorrelation Integral Power Ratio (IPR)

**Figure 2:**
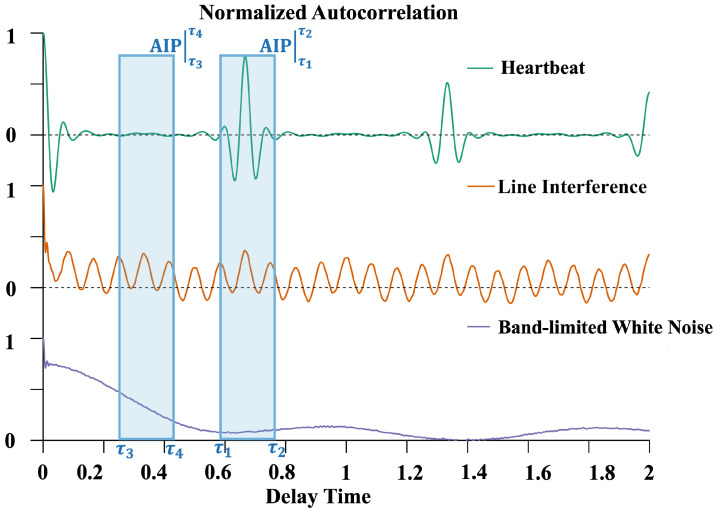
The autocorrelation function features of some components and the calculation method for the AIP.

The autocorrelation function of the mth independent component (IC) $C_{m}$ from ICA with respect to time lag *t* is expressed as (derivation provided in Appendix A):

(5) $$Ac_{\left(t\right)}\left(C_{m}\right)=\frac{1}{T-t}\frac{\sum_{i=1}^{T-t}\left(C_{m,i}-\bar{C_{m}}\right)\left(C_{m,i+t}-\bar{C_{m}}\right)}{\sigma_{m}^{2}}$$

where *T* denotes the total sequence length, and $\sigma_{m}^{2}$ represents the variance of component $C_{m}$. By analyzing this equation, several components and heartbeat signals can be characterized, as shown in Figure 2.

1. Bandwidth-limited Gaussian white noise (BWN): White noise generally exists in the system’s background noise. The bandwidth limitation of an OPM results in an autocorrelation function with sinc function characteristics, reflecting the time correlation introduced by the limited bandwidth.
2. Power line interference (PLI): these signals exhibit periodic characteristics in their autocorrelation functions.
3. Typical cardiac cycle: these signals have quasi-periodicity; the components should only have significant values when $t>T_{\min}$, where $T_{\min}$ is the shortest heartbeat period within the finite measurement time.

Based on these features, the variance of the autocorrelation function within a specified delay range around the heartbeat cycle can be used to calculate the autocorrelation integral power (AIP):

(6) $${\left.\mathrm{AIP}(m)\right|}_{\tau_{1}}^{\tau_{2}}=\mathrm{var}\left\{{\mathrm{Ac}}_{\left(t\right)}\left(C_{m}\right),\;t\in \left[-\tau_{1},\tau_{2}\right]\right\}$$

The AIP of an independent component with a dominant heartbeat signal is significantly different from that of a typical noise component. The IC dominated by the heartbeat signal should have lower AIP values. However, in real data, heartbeat-related signals often appear simultaneously in multiple ICs. The AIP distribution in different autocorrelation function delay intervals can be used to define the autocorrelation integral power ratio (IPR).

(7) $$\mathrm{IPR}\left(m\right)=\frac{{\left.\mathrm{AIP}(m)\right|}_{\tau_{1}}^{\tau_{2}}}{{\left.\mathrm{AIP}(m)\right|}_{\tau_{3}}^{\tau_{4}}}$$

The component with the largest IPR value can be selected as the template component for subsequent processing.

### 2.3. Cross-Correlation Function Interval Extremum

There exist unknown noise components whose autocorrelation function characteristics are not evident. These ICs are difficult to differentiate using the IPR. In such cases, the IC with the maximum IPR value is selected as the template sequence, denoted as follows:

(8) $$\mathrm{temp}=\left\{\mathrm{argmin}|\mathrm{IPR}(m)\right\}$$

Next, the bilateral cross-correlation function between this template sequence and other sequences $R_{\left(t\right)}\left(C_{\mathrm{temp}},C_{k}\right)$ is as follows:

(9) $$R_{\left(t\right)}\left(C_{temp},C_{k}\right)=\frac{1}{T-\left|t\right|}\cdot \frac{\sum_{i=1}^{T-\left|t\right|}\left(C_{temp,i}-\bar{C_{temp}}\right)\left(C_{k,i+t}-\bar{C_{k}}\right)}{\sigma_{temp}\cdot \sigma_{k}}$$

$\sigma_{temp}$ and $\sigma_{k}$ are the standard deviation of the two sequences, respectively.

Heartbeat signals exhibit quasi-periodic characteristics, meaning that each cardiac cycle has a similar repeating pattern, although the intervals between cycles are not fixed. Therefore, using periodic features to filter the target signal may not always be accurate. However, by leveraging quasi-periodic features, noise components (which do not exhibit cardiac cycle characteristics) can be filtered effectively.

**Figure 3:**
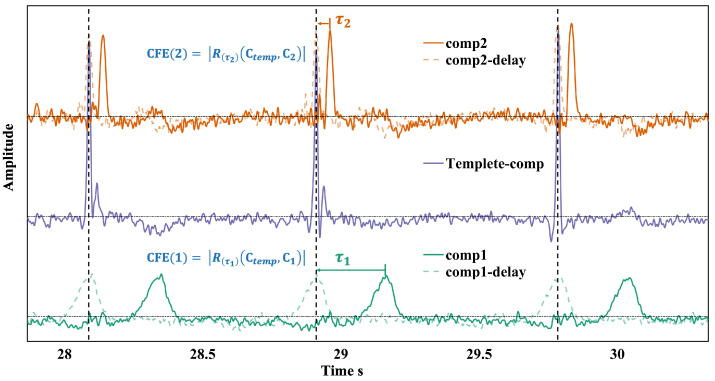
Template IC and CFE calculation for other components.

In practice, ICA often results in heartbeat signals appearing across multiple independent components (ICs). For example, the P-wave, QRS complex, and T-wave may be decomposed into different ICs. Without applying any time delay, the statistical properties of these components demonstrate independence. However, each cardiac cycle always includes the P-QRS-T complex, introducing a time delay, as shown in Figure 3. ICs containing any component of the P-QRS-T complex show a high degree of correlation.

In the delay range of $\pm \tau_{\mathrm{delay}}$, the extremum of the cross-correlation function is computed as:

(10) $$\mathrm{CFE}\left(k\right)=\max\left\{\left|R_{\left(t\right)}\left(C_{\mathrm{temp}},C_{k}\right)\right|\;|\;t\in \left[-\tau_{\mathrm{delay}},\tau_{\mathrm{delay}}\right]\right\}$$

The template $C_{\mathrm{temp}}$ exhibits the most pronounced cardiac cycle features. The value of $\tau_{\mathrm{delay}}$ is kept within half of the average cardiac cycle period, $T_{\mathrm{Avg}}$, to ensure that the delay allows the signals within the same cardiac cycle to overlap. Therefore, other ICs with cardiac cycle characteristics are evaluated by computing the CFE for each IC. The ICs dominated by cardiac cycle signals exhibit higher CFEs. ICs are then ranked based on their CFE values, and the top *N* ICs are retained, where *N* is determined by the variance contribution ratio estimated using Equation (3).

### 2.4. Methodology Flow

**Figure 4:**
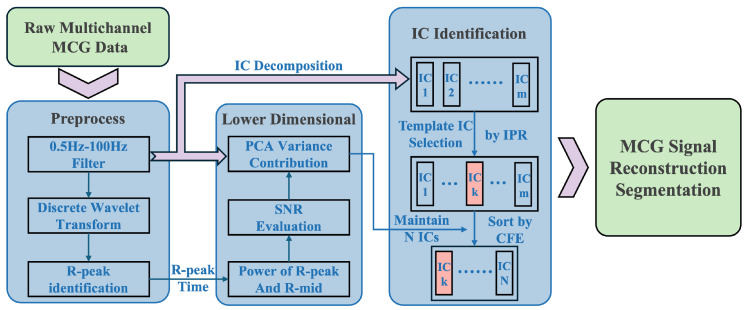
Flowchart of the adaptive ICA method.

The processing workflow of the adaptive ICA method is shown in Figure 4:

The raw data are passed through a bandpass filter (0.5 Hz to 100 Hz) to eliminate most of the baseline drift and high-frequency noise outside the sensor’s measurement bandwidth. Additionally, a 50 Hz spectral interpolation method is employed to remove PLI from the environment. The resulting data undergo discrete wavelet decomposition, extracting the wavelet coefficients from the frequency band (5–40 Hz) where the R-wave energy is concentrated, and reconstructing the signal using three layers of wavelet coefficients.

The R-wave localization algorithm is used to identify the location of each R-wave. The signal is then segmented into epochs, and the initial SNR is estimated.

The preprocessed multi-channel data undergo PCA, which is used to estimate the variance contribution threshold of PCA under the given SNR conditions. This information is used to calculate the number of principal components to retain, thereby determining the target dimensionality (N) for reducing the dimensionality of the multi-channel data.

Next, ICA is applied to decompose the original multi-channel data. Based on the IPR, the template IC is selected. The CFE between the template IC and other ICs is calculated, and all the ICs are ranked according to their CFE values. The top N ICs are retained and recombined, completing the adaptive ICA process for OPM-MCG.

## 3. Experiment

### 3.1. Phantom Experiment

**Figure 5:**
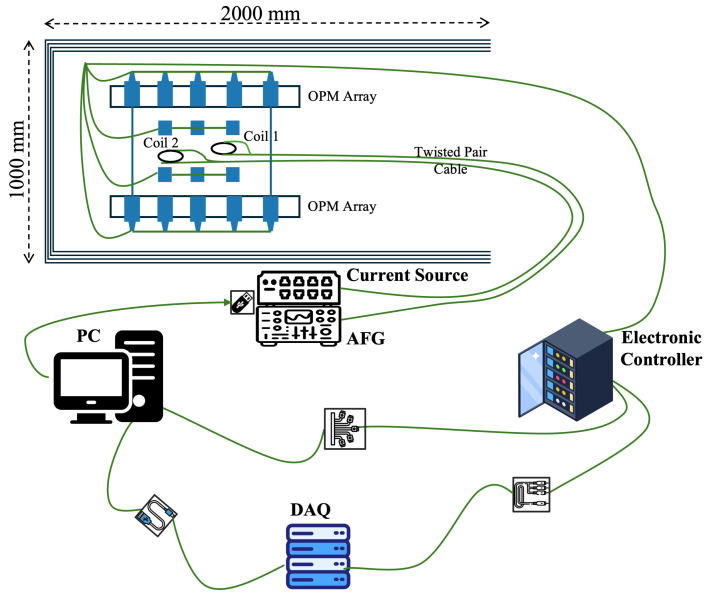
Phantom simulation system setup.

The setup for the physical simulation experiment system is shown in Figure 5.

The simulated heartbeat signals were generated using the Python 3.9 package neurokit2. The generated heartbeat signals included modulation effects caused by respiration and certain heart rate variability. The heart rate was set to 90 bpm, the data length was 100 s, and the sampling rate was 1 kHz. This signal served as the true heartbeat signal to be recovered.

**Figure 6:**
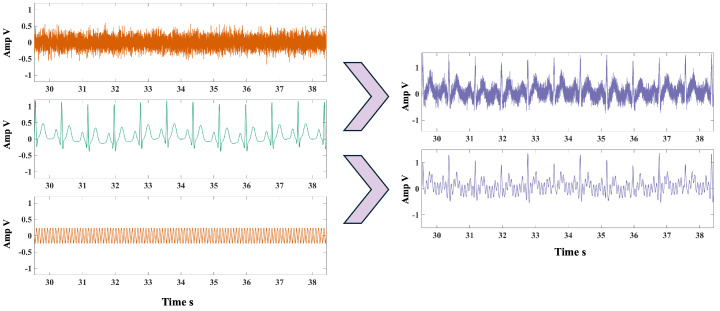
Simulated heartbeat signal (green), interference signal waveform (orange), and synthetic signal (purple).

As shown in Figure 6, the interference signals consisted of PLI signals with varying amplitudes and BWN signals. The frequency of the PLI signal was set to 12 Hz, overlapping with the heartbeat signal’s frequency band. The initial SNR was set to 0 dB, 3 dB, 5 dB, and 7 dB, based on the ratio of the power of the simulated heartbeat signal to the interference signals.

The coils of the magnetic dipoles were fixed in the OPM array, which was distributed across four planes: upper, lower, left, and right. A total of 64 OPM sensors were configured. The experiment was conducted within a magnetic shielding barrel made of four layers of Permalloy, with a single-ended closed cover and an active lid, and the DC residual magnetism inside the barrel was 2 nT. The magnetic field signals output from the OPMs were passed through a magnetometer controller array and sent to a synchronized DAQ system. The analog voltage signals were sampled with a 16-bit AD sampling precision at a rate of 1.2 kS/s. The conversion ratio from the measured magnetic field to the analog voltage signal was set to 2.7 V/nT. The test data were collected and stored through an upper-level computer for subsequent offline processing.

During the experiment, once the sensors were activated, both dipole coils were simultaneously energized, and the magnetic field in the space was superimposed with both the heartbeat and noise signals. The sensor array recorded the mixed magnetic field signals in the space. Using the ICA algorithm, the components containing the heartbeat signals, PLI signals, and white noise were separated. The proposed artifact component identification method was used to automatically screen the IC components. The coefficient of the correlation between the reconstructed components and the original heartbeat signal, as well as the output SNR of the array, were calculated to assess the algorithm’s signal recovery and interference suppression capability.

### 3.2. MCG Data Acquisition

To verify the applicability of the method proposed in this study and compare its efficiency in signal processing, heartbeat signals from a real human subject were collected and processed.

The subject was a male with normal heart function. During the human experiment, the subject was placed on a test bed inside the magnetic shielding barrel, with sensors embedded around the bed. The shielding barrel isolated the environmental magnetic field to minimize interference with the test. It also provided an environment with a nearly zero magnetic field for the atomic magnetometer. The shielding barrel remained open at one end throughout the test. Each volunteer was informed of the experiment and gave their consent. During each data collection session, the subject was instructed to stay awake and relaxed to avoid heart rate abnormalities caused by tension. The test duration was 90 s.

The collected data were processed using the method proposed in this study, and the processing time was compared with the time taken by seven experienced data processing operators to select artifact IC components manually. The time required to select the ICs was calculated to verify the efficiency improvement of the proposed method for real MCG signals.

## 4. Results

### 4.1. Phantom Experiment

**Figure 7:**
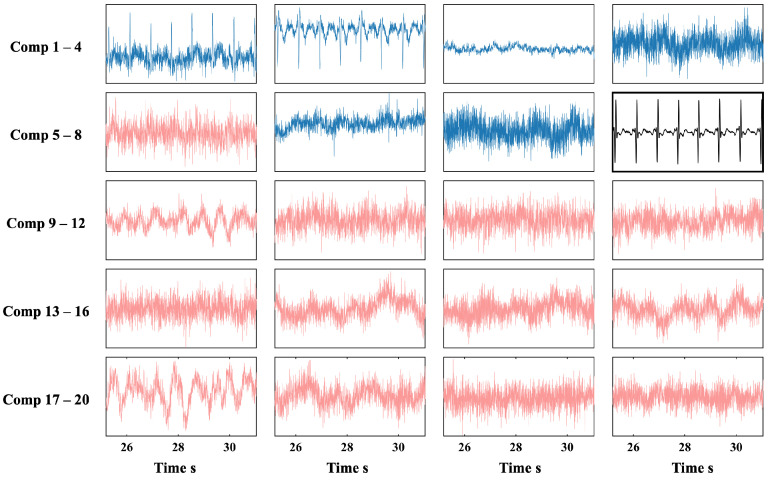
Independent component waveforms and screening results for phantom simulation experiments. The ICs to be excluded are in red, the retained ICs are in blue, and the template IC is in black.

**Figure 8:**
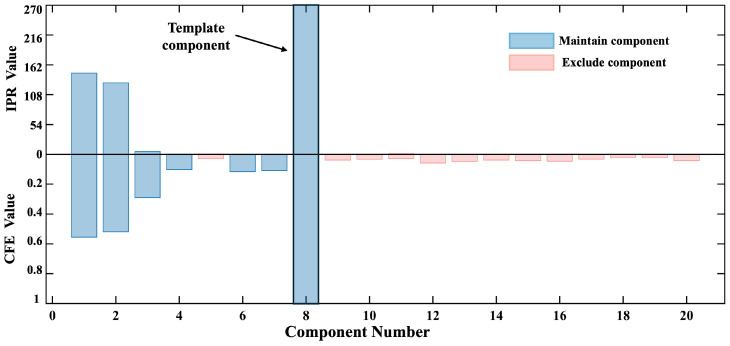
IPR and CFE values of the independent components extracted from the simulated signal.

Figure 7 shows the ICs extracted from the original 128-channel OPM-MCG data after ICA decomposition, with 3 dB of BWN. The results included 20 independent components. The IPR values and CFE calculation results for all the components are shown in Figure 8. The black component (comp 8) was selected as the template component based on the maximum IPR, with an IPR value of 270, indicating that this component had an AIP 270 times greater than that in the delay interval of interest, making it suitable as a template component for subsequent filtering.

The initial SNR estimate for the array calculated the proportion of signal variance to total variance, which was then used as the threshold for PCA dimensionality reduction. The calculations showed that seven principal components (N = 7) contributed to the estimated SNR corresponding to the required variance contribution rate.

The CFEs of all the ICs (calculated from zero to one) were sorted, and the top N independent components were retained, while the remaining ICs were identified as non-heartbeat-related artifacts. This allowed the automatic filtering of the ICs. The retained components were multiplied by the demixing matrix to reconstruct the multi-channel signal, completing the denoising of the original 128-channel signal.

**Figure 9:**
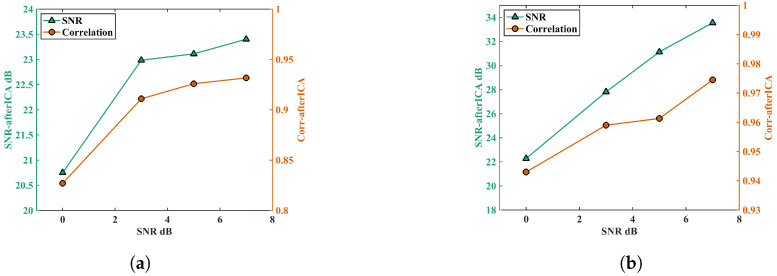
Total output SNR of all channels after processing of the initial simulated signal with different input SNR (left coordinate), correlation coefficient between the recovered heartbeat signal and the input simulated heartbeat signal (right coordinate) (**a**) Interfering signal is PLI (**b**) Interfering signal is BWN.

Using this processing method, signal cleaning was performed on different types of interference signals and input SNRs. The processed results are shown in Figure 9.

Phantom experiments data were processed using adaptive ICA. Figure 9 illustrates the output SNR (${\mathrm{SNR}}_{\mathrm{out}}$) after processing under different types of interference signals and various input SNRs (${\mathrm{SNR}}_{\mathrm{in}}$), as well as the correlation coefficient between the recovered heartbeat and input simulated heartbeat signal.

Figure 9a shows that, when the input signal was the simulated heartbeat signal with added PLI noise, as the input SNR increased (i.e., the power of the added interference signal decreased), the SNR of the output multi-channel signal improved. At an input SNR of 7 dB, the output SNR reached up to 23.5 dB. The SNR improvement ratio (${\mathrm{SNR}}_{\mathrm{out}}-{\mathrm{SNR}}_{\mathrm{in}}$) decreased gradually, indicating that the proposed method performed better in suppressing noise when the input signal had higher noise levels.

Figure 9b shows the results when the input is the simulated heartbeat signal with added BWN. Compared to the PLI, the algorithm showed better performance in suppressing BWN. The SNR improvement ratio for different input SNRs of BWN interference was relatively consistent.

As the input SNR increased, the coefficient of the correlation between the reconstructed signal and the original input signal also rose. At an input SNR of 7 dB with BWN interference, the coefficient of the correlation between the output signal and the input simulated heartbeat signal reached 0.975.

### 4.2. Real MCG Data

**Figure 10:**
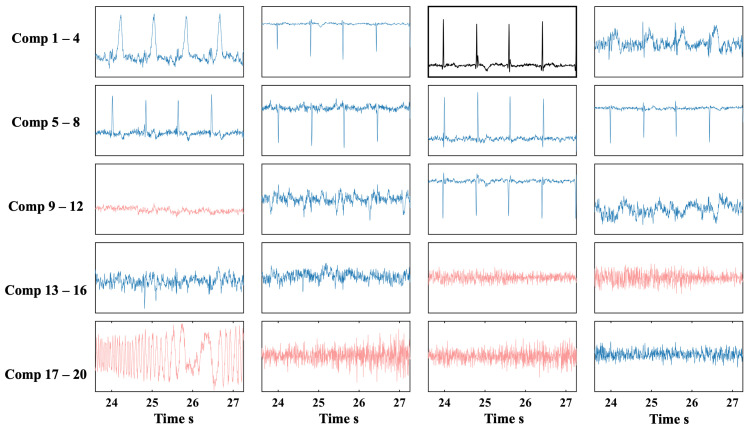
Independent components and their identification for real MCG signals processed using the adaptive ICA method.The ICs to be excluded are in red, the retained ICs are in blue, and the template IC is in black.

Figure 10 shows the ICA decomposition results for real MCG signals. Compared to the physical simulation results, the decomposition of actual MCG signals was more complex, with heartbeat-related signals appearing in multiple ICs, and different waves of a heartbeat cycle, such as the P wave, QRS complex, and T wave, being decomposed into different ICs.

**Figure 11:**
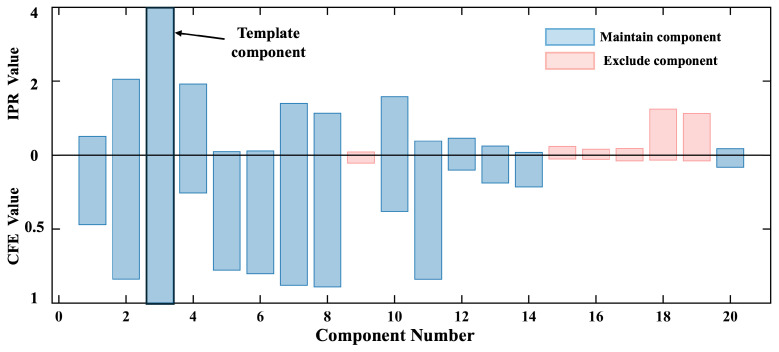
IPR and CFE values of independent components extracted from real MCG.

Figure 11 shows the IPR and CFE values for each IC. Based on the IPR, comp 3 was selected as the template component. The initial SNR estimate from PCA dimensionality reduction indicated that 14 components (N = 14) needed to be retained. This indicated that the initial SNR of real MCG signals was better than that of the simulated signal.

**Figure 12:**
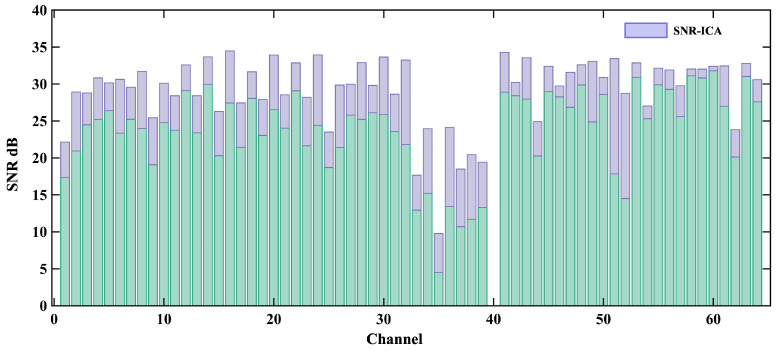
SNR of channels 1 to 64 and the SNR improvement ratio achieved using the adaptive ICA method.Green bar is the SNR before adaptive ICA.

**Figure 13:**
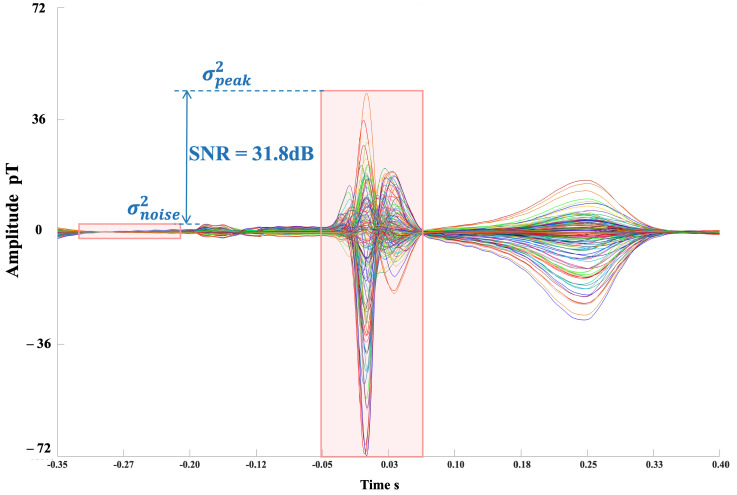
Butterfly plot and total array SNR for all channels after signal reconstruction.

Figure 12 shows the output SNR for the first 64 channels, with the contribution of the ICA algorithm to the overall SNR improvement. After processing, most of the channels in the real MCG signal showed an output SNR greater than 30 dB. The total array SNR was calculated to be 31.8 dB. Figure 13 shows the butterfly plot of the reconstructed signals for all the channels.

The comparison of the processing time between the proposed method and the manual processing by seven experienced operators is summarized in Table 1. “Excluded components” refer to the components excluded from the decomposed independent components based on the empirical judgment of each experimenter. “Time cost” represents the time required for each experimenter to perform the component selection process. “SNR_IMP” denotes the SNR improvement (${\mathrm{SNR}}_{\mathrm{out}}-{\mathrm{SNR}}_{\mathrm{in}}$) after excluding the components.

The results show that, even among experienced operators, there were still differences in the results. The average processing time was about 69 s. The proposed adaptive ICA method requires only 1.8 s for IC selection. The SNR_IMP results indicate that our method achieved the highest SNR improvement.

## 5. Discussion

MCG signals are usually exposed to severe environmental and physiological signal contamination, and blind source separation of the signal using ICA is a common processing method. However, component identification in ICA still relies heavily on human intervention. Different experimenters may have subjective judgments regarding whether a component is an artifact or not, leading to variability in the results and making it difficult to perform quantitative evaluations. Manual involvement in the process also adds complexity and increases the processing time required.

In this study, we introduced the use of the time-domain correlation features of MCG signals, specifically the autocorrelation integral power ratio (IPR) and cross-correlation function extremum (CFE) metrics, to quantitatively evaluate and select ICs. Through phantom simulation experiments, the effectiveness of this method was validated. Additionally, real MCG signals were tested, and the results demonstrated good IC identification performance.

It is worth noting that the proposed adaptive ICA method was validated for applications in multi-channel OPM-MCG; however, this does not imply that the method is exclusively applicable to OPM-MCG. ICA is also commonly used for artifact suppression in SQUID-MCG signals [22,23]. This suggests that, with appropriate adaptations and minor modifications, the proposed method could also be effective in SQUID-MCG.

### 5.1. Comparison with Previous Work

With the gradual maturation and commercialization of magnetic field sensor technology, particularly OPM-MCG systems, research on MCG has been steadily increasing. Simultaneously, the demand for efficient signal processing workflows has become more urgent. Due to the flexible spatial arrangement of OPM-MCG systems and the complexity of the magnetic field environment, MCG signal processing faces new challenges.

Earlier studies used methods combining empirical mode decomposition (EMD) or its derivative, ensemble EMD (EEMD), with ICA for noise reduction and analysis, which showed some success in improving signal quality [18,24,25]. These methods increased the computational complexity by first expanding the data in a single channel before applying ICA for dimensionality reduction [26], which became more complicated, especially when dealing with multiple channels. More recent MCG studies have increasingly adopted wavelet decomposition combined with ICA due to the need to superimpose heartbeat signals across cycles [27].

This study observed that the least portable part of the ICA calculation process was the manual selection of ICs. Due to the unique quasi-periodic nature of MCG signals, the autocorrelation and cross-correlation functions of the ICs related to the heartbeat showed distinct characteristics. This enabled the ICA algorithm in MCG signal processing to use the IPR and CFE for the more quantitative selection of ICs. Compared to those of previous methods, the accuracy and efficiency of IC selection were significantly improved.

### 5.2. Performance on Different Input Signals

When comparing the performance on two types of simulated signals, the adaptive ICA method demonstrated superior noise suppression for BWN compared to PLI. This was reflected in the comparison of the SNR improvement ratios and correlation coefficients. As shown in Figure 9a,b, under the same input SNR of 7 dB, the adaptive ICA method produced a final SNR of 33 dB with BWN, with a correlation coefficient close to 0.98. In contrast, when PLI was present, the final SNR reached only 23 dB, with a correlation coefficient of 0.93. This difference likely arises from two factors: (1) the accuracy of the SNR estimation differs for the two types of interference, which affects the PCA dimensionality reduction process for determining the number of retained components; (2) ICA has a lower ability to distinguish sine-like signals such as PLI.

When comparing the simulated and real MCG data, we observed that the number of components retained for real data was significantly larger than that for simulated signals. This is because the simulated heartbeat signals were generated by a coil, and their spatial distribution only involved amplitude differences between channels. In contrast, the magnetic fields generated by actual cardiac electrophysiological activity cannot be fully described by a single magnetic dipole. A real heartbeat signal exhibits amplitude differences and phase shifts among different sensors, requiring more independent components to adequately describe the signal in OPM-MCG data processing.

### 5.3. Parametric Analysis

**Figure 14:**
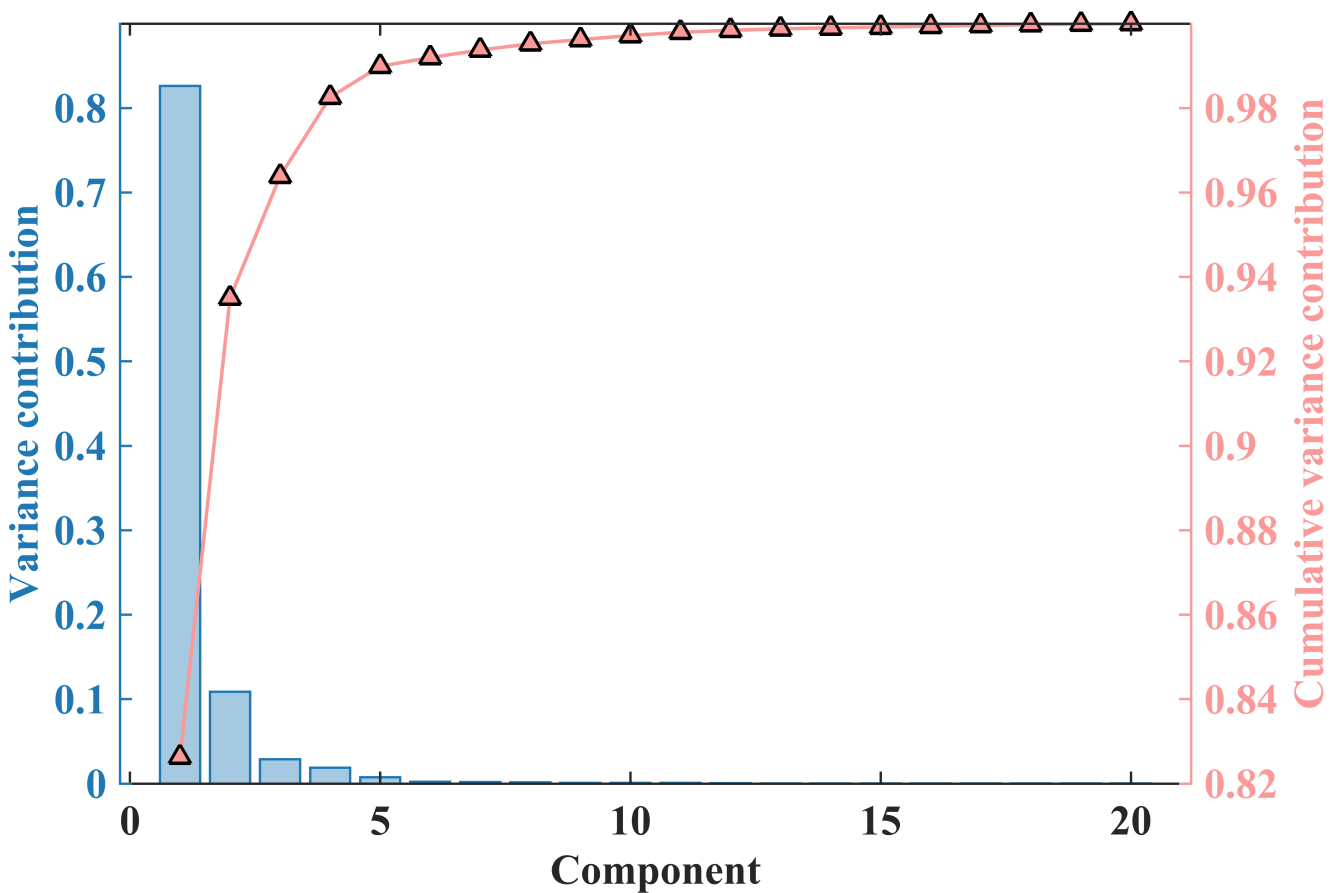
Variance and cumulative contribution to variance of each principal component from PCA decomposition.

Equation (4) represents the process of determining the number of principal components in PCA dimensionality reduction based on the variance contribution rate. This process involves computing the variance of each component obtained through PCA decomposition and ranking them in descending order. The number of retained components is determined when the cumulative variance contribution rate of the first N components exceeds a given threshold. Figure 14 illustrates the variance of PCA components and the cumulative variance contribution rate.

Under high SNR conditions, the selection of retained components is highly sensitive to the chosen threshold. However, in previous approaches, this threshold was often selected empirically, varying across different testing systems and environments. In this study, the SNR of the input signal was pre-evaluated, and the threshold was estimated based on that SNR. This estimation method enhances the objectivity and robustness of the processing procedure.

Equation (7) for IPR estimation, which involved parameters such as $\tau_{1}$ and $\tau_{2}$, was addressed in the preprocessing stage. Specifically, R-peaks were first detected, and the time intervals between consecutive R-peaks were computed as individual heartbeat intervals. The durations of all heartbeat intervals within the testing period were then calculated, where $\tau_{1}$ and $\tau_{2}$ represented the shortest and longest intervals, respectively. Within this delay range, the contribution of each heartbeat to AIP computation could be accumulated throughout the testing period.

In the calculation of the CFE, the parameter $\tau_{\mathrm{delay}}$ corresponded to the average heartbeat interval within the testing period. The cross-correlation maximum was determined within the range of $\pm \tau_{\mathrm{delay}}$. Since the cross-correlation maxima among different independent components typically occurred within the same heartbeat cycle, the CFE calculation was not highly sensitive to the selection of $\tau_{\mathrm{delay}}$.

### 5.4. Limitations

Although the method proposed in this study was thoroughly validated under the current noise levels in the system, there are some limitations and important considerations regarding its application and underlying principles.

The proposed adaptive ICA processing method exhibits potential applicability in the diagnosis of various clinical cardiac diseases, such as coronary artery disease, arrhythmias, and myocardial ischemia [3,28,29]. However, it is important to note that if a subject presents electrophysiological characteristics associated with non-sinus rhythms, such as ectopic pacemakers, manual intervention may still be required during processing. Future research will further explore data collection and feature extraction for such cardiac conditions, integrating these aspects with existing methods to expand the applicability of the proposed approach.

Additionally, the use of OPMs to measure cardio-magnetic signals in unshielded environments has received increasing attention. In such measurements, the number of available OPM sensors is limited, making the proposed multi-channel processing method unsuitable for these applications.

## 6. Conclusions

As OPM-MCG technology becomes more widely applied in clinical settings, there is an increasing demand for signal processing methods that can be adapted to a wide range of scenarios. ICA has shown significant denoising effects in MCG signal processing. In this study, based on the ICA algorithm, we proposed an improved adaptive ICA method for multi-channel OPM-MCG signal processing. This method was validated using both simulated signals and real MCG data.

Further research in this area could involve expanding the sample size and incorporating large data models for training. By leveraging more dimensional features, such as spatial data, to distinguish between noise and signal, the artifact identification and noise suppression capabilities could be further enhanced.

This study has made an important step toward solving key issues in OPM-MCG signal processing. The results indicate that the proposed method provides strong support for the broader clinical application and further development of OPM-MCG technology in the field of cardiology.

## Figures and Tables

**Table 1:** Comparison of processing time and results between the adaptive ICA algorithm and manual processing by experienced operators.

	Excluded Components	Time Cost (s)	SNR_IMP (dB)
1	9, 10, 13, 14, 15, 16	58	2.72
2	9, 10, 11, 13, 14, 15, 16	48	3.27
3	9, 10, 11, 13, 15, 16	121	3.24
4	9, 10, 11, 13, 14, 15, 16	33	3.27
5	9, 10, 11, 14, 15, 16	89	2.92
6	9, 10, 14, 15, 16	28	2.70
7	6, 9, 10, 11, 13, 14, 15, 16	107	3.07
**Adaptive ICA**	9, 10, 11, 13, 14, 15, 16	1.8	3.27

## Data Availability

Data will be made available on request.

## Appendix A

This Appendix A is a detailed derivation of the autocorrelation function for the Section 2.

For two continuous real signals x(t) and y(t), the cross-correlation function with respect to time delay τ is defined as:

(A1) $$R_{xy}\left(\tau \right)=\int_{-\infty }^{\infty }x\left(t\right)y\left(t+\tau \right)dt$$

For discrete sampled time sequences x(n) and y(n), the cross-correlation function at delay sampling point *t* is given by:

(A2) $$R_{xy}\left(t\right)=\sum_{n=-\infty }^{+\infty }x\left(n\right)y\left(n+t\right)$$

Since practical signals are of finite length, assuming the time sequence length is *T*, the normalized finite-length discrete cross-correlation function is expressed as:

(A3) $$R_{xy}\left[t\right]=\frac{\sum_{n=1}^{T-t}\left(x\left[n\right]-\mu_{x}\right)\left(y\left[n+k\right]-\mu_{y}\right)}{\sqrt{\sum_{n=1}^{T-t}{\left(x\left[n\right]-\mu_{x}\right)}^{2}}\cdot \sqrt{\sum_{n=1}^{T-t}{\left(y\left[n\right]-\mu_{y}\right)}^{2}}}$$

where $\mu_{x}$$\mu_{y}$ represents the mean of the overlapping segments of the two sequences at delay *t*:

(A4) $$\mu_{x}=\frac{1}{T-t}\sum_{n=1}^{T-t}x\left[n\right],\;\mu_{y}=\frac{1}{T-t}\sum_{n=1}^{T-t}y\left[n\right]$$

and the variance of the overlapping segments is given by:

(A5) $$\sigma_{x}=\sqrt{\frac{1}{T-t}\sum_{n=0}^{T-t}{\left(x\left[n\right]-\mu_{x}\right)}^{2}},\sigma_{y}=\sqrt{\frac{1}{T-t}\sum_{n=0}^{T-t}{\left(y\left[n\right]-\mu_{y}\right)}^{2}}$$

Thus, the normalized cross-correlation function can be simplified as:

(A6) $$R_{xy}\left[t\right]=\frac{1}{T-t}\cdot \frac{\sum_{n=1}^{T-t}\left(x\left[n\right]-\mu_{x}\right)\left(y\left[n+k\right]-\mu_{y}\right)}{\sigma_{x}\cdot \sigma_{y}}$$

If x(n) and y(n) are two independent components obtained via independent component analysis (ICA), denoted as $C_{m}$ and $C_{n}$, respectively, the cross-correlation function is given by:

(A7) $$R_{\left(t\right)}\left(C_{m},C_{n}\right)=\frac{1}{T-t}\cdot \frac{\sum_{i=1}^{T-t}\left(C_{m,i}-\bar{C_{m}}\right)\left(C_{n,i+t}-\bar{C_{n}}\right)}{\sigma_{m}\cdot \sigma_{n}}$$

where $\bar{C_{m}}$ and $\bar{C_{n}}$ represent the mean of the overlapping segments of the two components.

In particular, when m=n, the cross-correlation function degenerates into the autocorrelation function of the component at delay *t*:

(A8) $$Ac_{\left(t\right)}\left(C_{m}\right)=\frac{1}{T-t}\frac{\sum_{i=1}^{T-t}\left(C_{m,i}-\bar{C_{m}}\right)\left(C_{m,i+t}-\bar{C_{m}}\right)}{\sigma_{m}^{2}}$$

In another case, when discussing the bilateral cross-correlation function of two sequences, the delay *t* can take negative values. In this scenario, the cross-correlation function can be expressed as:

(A9) $$R_{\left(t\right)}\left(C_{m},C_{n}\right)=\frac{1}{T-\left|t\right|}\cdot \frac{\sum_{i=1}^{T-\left|t\right|}\left(C_{m,i}-\bar{C_{m}}\right)\left(C_{n,i+t}-\bar{C_{n}}\right)}{\sigma_{m}\cdot \sigma_{n}}$$
